# Supplementary material for: Evanescent field trapping and propulsion of Janus particles along optical nanofibers
Source: Nat Commun. 2023 Mar 27;14:1691. doi: 10.1038/s41467-023-37448-2 (PMC10043011; doi:10.1038/s41467-023-37448-2)
Supplement: Supplementary file 1 — Supplementary Information [file 41467_2023_37448_MOESM1_ESM.pdf]

Supplementary information
Evanescent field trapping and propulsion of Janus particles along optical nanofibers

Georgiy Tkachenko,* Viet Giang Truong, Cindy Liza Esporlas, Isha Sanskriti, and Síle Nic Chormaic†
Okinawa Institute of Science and Technology Graduate University, Onna, Okinawa 904-0495, Japan

Supplementary Note 1. Optomechanical simulations

Supplementary Figure 1a shows the theoretical model we used for a spherical silica particle (SP) or Janus particle (JP, represented by a SP half-coated with gold) interacting with the evanescent light field near a single-mode optical nanofiber (ONF). Once the field distributions and the Maxwell's stress tensor, $\overleftrightarrow{T}$, over the computational volume are found (see Methods section in the main text), we perform surface integration (directly in COMSOL) of the elementary force, $d\mathbf{F}$, and torque, $d\mathbf{N}$, presented as

$$d\mathbf{F} = \hat{n}\cdot \overleftrightarrow{T}\, dS = \begin{pmatrix} \mathrm{T}_x \\ \mathrm{T}_y \\ \mathrm{T}_z \end{pmatrix} , \tag{S.1}$$

$$d\mathbf{N} = -\hat{n}\cdot\left(\overleftrightarrow{T}\times\mathbf{r}\right) dS = -\begin{vmatrix} \mathbf{x} & \mathbf{y} & \mathbf{z} \\ \mathrm{T}_x & \mathrm{T}_y & \mathrm{T}_z \\ x & y & z \end{vmatrix} = -\begin{pmatrix} z\,\mathrm{T}_y - y\,\mathrm{T}_z \\ x\,\mathrm{T}_z - z\,\mathrm{T}_x \\ y\,\mathrm{T}_x - x\,\mathrm{T}_y \end{pmatrix} , \tag{S.2}$$

where $\mathrm{T}_x = -$ewbe.dnTx, $\mathrm{T}_y = -$ewbe.dnTy, $\mathrm{T}_z = -$ewbe.dnTz are the COMSOL expressions for the x, y, and z components of the "Maxwell downward (i. e. *inwardly* directed) surface stress tensor", respectively. The "−" sign in the expressions for $\mathrm{T}_{x,y,z}$ appears because we follow [1] and define the normal unit vector, $\hat{n}$, as *outwardly* directed with respect to the surface element, dS.

As shown in Supplementary Figure 1b, only the x component of the optical torque on a JP is nonzero, $\mathbf{N} = \mathbf{N}_x = \mathbf{x}N_x \neq 0$ (more precisely, $|N_x/N_y| \approx |N_x/N_z| \gtrsim 10^5$), hence the particle can only spin in the yz plane. In fact, we

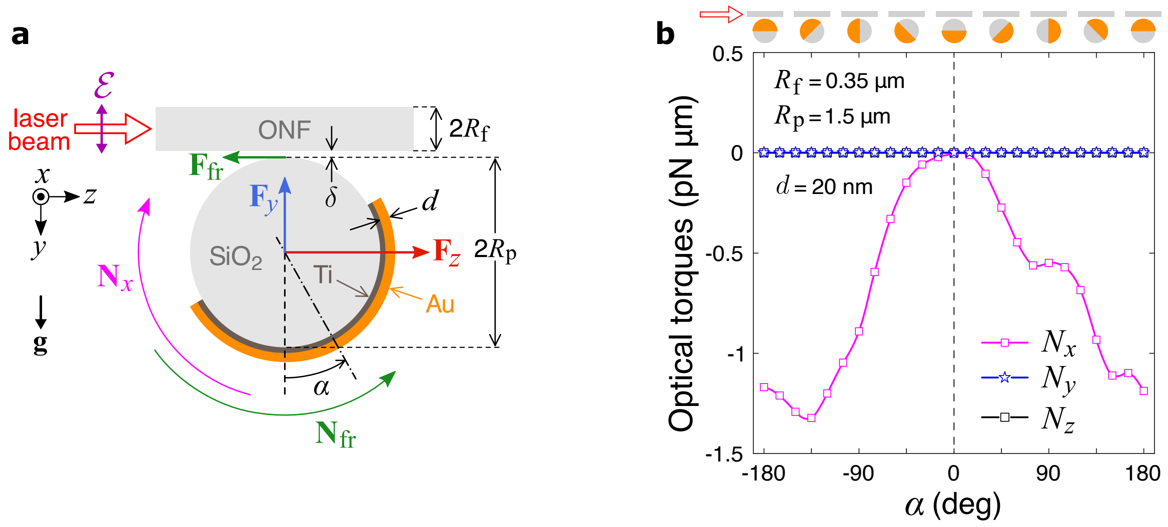

Supplementary Figure 1. **a** Model schematics, where $\mathcal{E}$ is the electric field vector; $\mathbf{g}$ is the gravitational acceleration; $\mathbf{N}_x$ is the optical torque; $\mathbf{N}_{\mathrm{fr}}$ is the torque produced by the friction force, $\mathbf{F}_{\mathrm{fr}}$; $\mathbf{F}_z$ and $\mathbf{F}_y$ are the longitudinal and radial optical forces, respectively. **b** Simulated components of the optical torque versus the gold cap orientation. The guided mode has a vacuum wavelength of $\lambda = 1.064\,\mu$m and a power of $P = 1$ mW. Markers indicate the simulated points, lines refer to spline interpolations. Sketches on top show the gold cap orientation at every 45 degrees.

* email: georgiy.tkachenko@oist.jp
† email: sile.nicchormaic@oist.jp

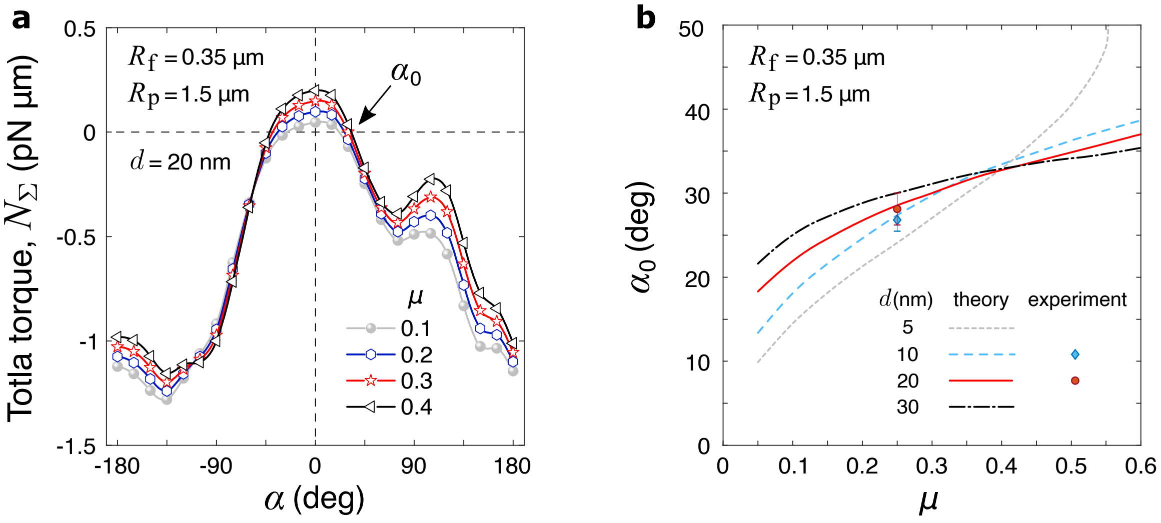

Supplementary Figure 2. **a** Total torque on a Janus particle calculated using Eq. S.3 with different values of the friction coefficient, μ. The balance orientation angle, α_0, increases with μ. **b** Balance orientation angle, α_0, versus the friction coefficient for different coating thickness, d. Markers (placed at $\mu = 0.25$ corresponding to the best fit between the theory and the experiment) indicate the measured values of α_0. Error bars in **b** are the same as in Fig. 3c in the main text.

get $N_x < 0$ for any orientation angle of a JP, α, and this result agrees with the rotation one would expect from the direction-locked transverse spin angular momentum (SAM) carried by the evanescent field [2, 3]. It is worth noting that if the ONF mode broke the mirror symmetry of the system, we should expect to see the impact of the other torque components. In particular, in the case of a circularly polarized input beam, the JP would experience $\mathbf{N}_z \neq 0$ due to the longitudinal SAM of light. In the case of a tilted linear polarization, a vertical "electric" component of SAM would appear [4], forcing the particle to rotate around the y axis under $\mathbf{N}_y \neq 0$. However, in practice, this rotation would be transient and would only occur while the JP is being pulled into the higher-intensity region (that is the polarization plane) of the field, before settling at a certain orientation defined by the optical forces, gravity, and friction.

Now let us discuss the dynamic friction force, $\mathbf{F}_{\rm fr}$. It opposes the propulsion of the particle when it is pressed to the ONF by the radial optical force, $\mathbf{F}_y$. Since $\mathbf{F}_{\rm fr}$ is applied at the particle-nanofiber contact point, it produces a torque, $\mathbf{N}_{\rm fr} = R_{\rm p}\mathbf{y} \times \mathbf{F}_{\rm fr}$, which counteracts the optical torque, $\mathbf{N}_x$. The total torque acting on a JP is therefore

$$N_\Sigma = N_x + N_{\rm fr} = N_x - \mu F_y R_{\rm p}\,, \tag{S.3}$$

where $\mu \in [0, 1]$ is the friction coefficient. Under the action of N_Σ, the particle keeps rotating until it reaches a balance orientation at an angle α_0, which corresponds to $N_\Sigma = 0$ and $dN_\Sigma/d\alpha < 0$ (the restorative torque condition). As shown in Supplementary Figure 2a, α_0 increases with μ. In this work, we find μ by fitting the theoretical α_0 values (obtained via spline interpolation of the N_Σ points calculated using Eq. S.3) to those measured experimentally (see Supplementary Note 3). The best fit for 10- and 20-nm-thick gold coatings corresponds to $\mu = 0.25$ (Supplementary Figure 2b), a realistic value [5, 6] which we keep constant for the subsequent simulations of forces and torques on JPs near ONFs of different thicknesses (Fig. 5 in the main text).

In addition, we check if the variability of $R_{\rm p}$ (particle radius) and δ (particle-nanofiber separation) could have a significant impact on our results. These parameters could not be measured directly using our experimental approach, hence we performed test simulations with $R_{\rm p} = \{1.4, 1.5, 1.6\}\,\mu$m (since $2R_{\rm p} = 3.13 \pm 0.20\,\mu$m, according to the manufacturer) and $\delta = \{10, 100, 150\}$ nm. As one can see in Supplementary Figure 3, theoretical curves for the optical forces and total torques values at variable $R_{\rm p}$ (and fixed $\delta = 10$ nm) or variable δ (and fixed $R_{\rm p} = 1.5\,\mu$m) are somewhat different; yet, the balance orientation of the gold cap, α_0, is almost unchanged, and the corresponding values of F_z (and the propulsion enhancement, ξ_z) vary by less than 2%. This uncertainty is about ten times smaller than the standard deviation ranges in our experiments. Therefore, we believe that the main source of error was the nanoscale variability of the gold coating.

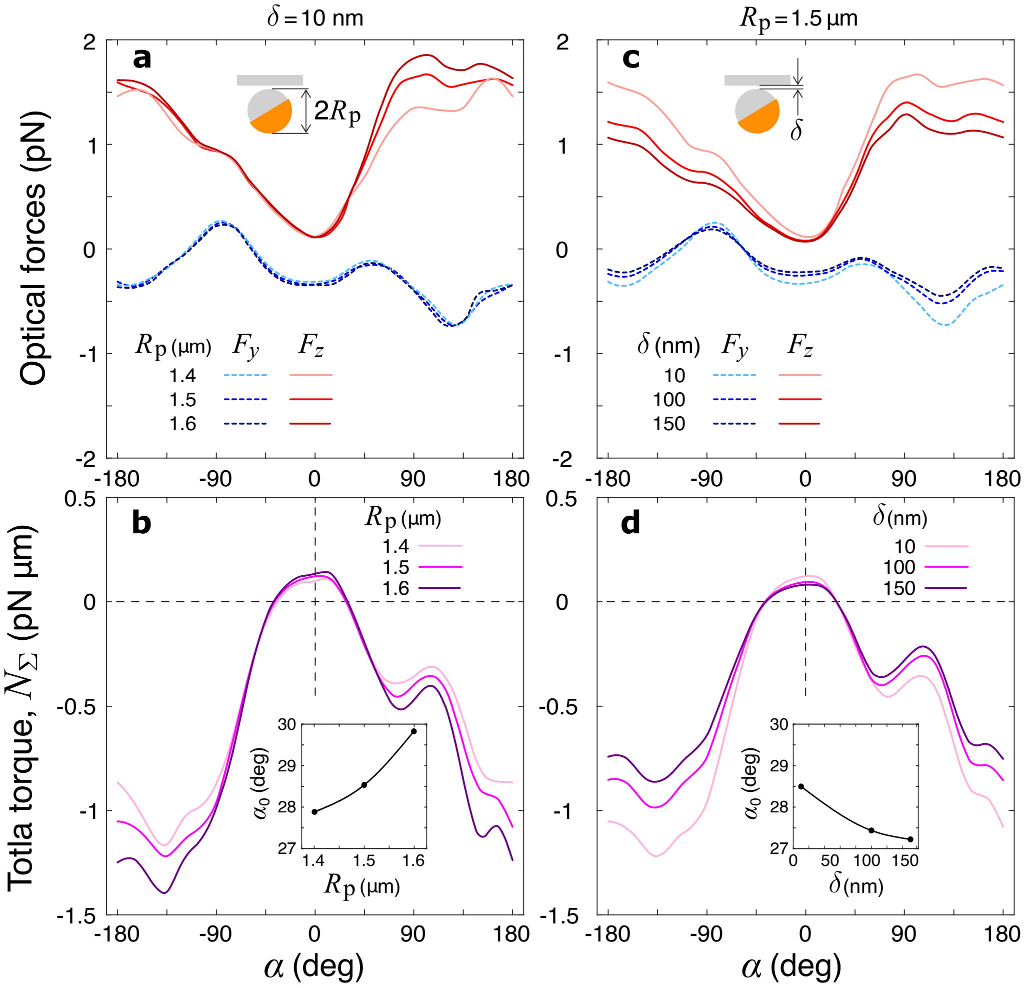

Supplementary Figure 3. **a, b** Radial and longitudinal optical forces (**a**) and the total torque (**b**) on a Janus particle with a variable radius, R_p, and a fixed distance to the nanofiber, $\delta = 10$ nm. **c, d** Same for a variable δ and fixed radius, $R_\mathrm{p} = 1.5\,\mu$m. Insets in **c, d**: balance orientation of the golden cap (found at $N_\Sigma = 0$) vs. the particle radius and the particle-nanofiber separation distance, respectively. In all simulations we used $R_\mathrm{f} = 0.35\,\mu$m, $d = 20$ nm, and $\mu = 0.25$.

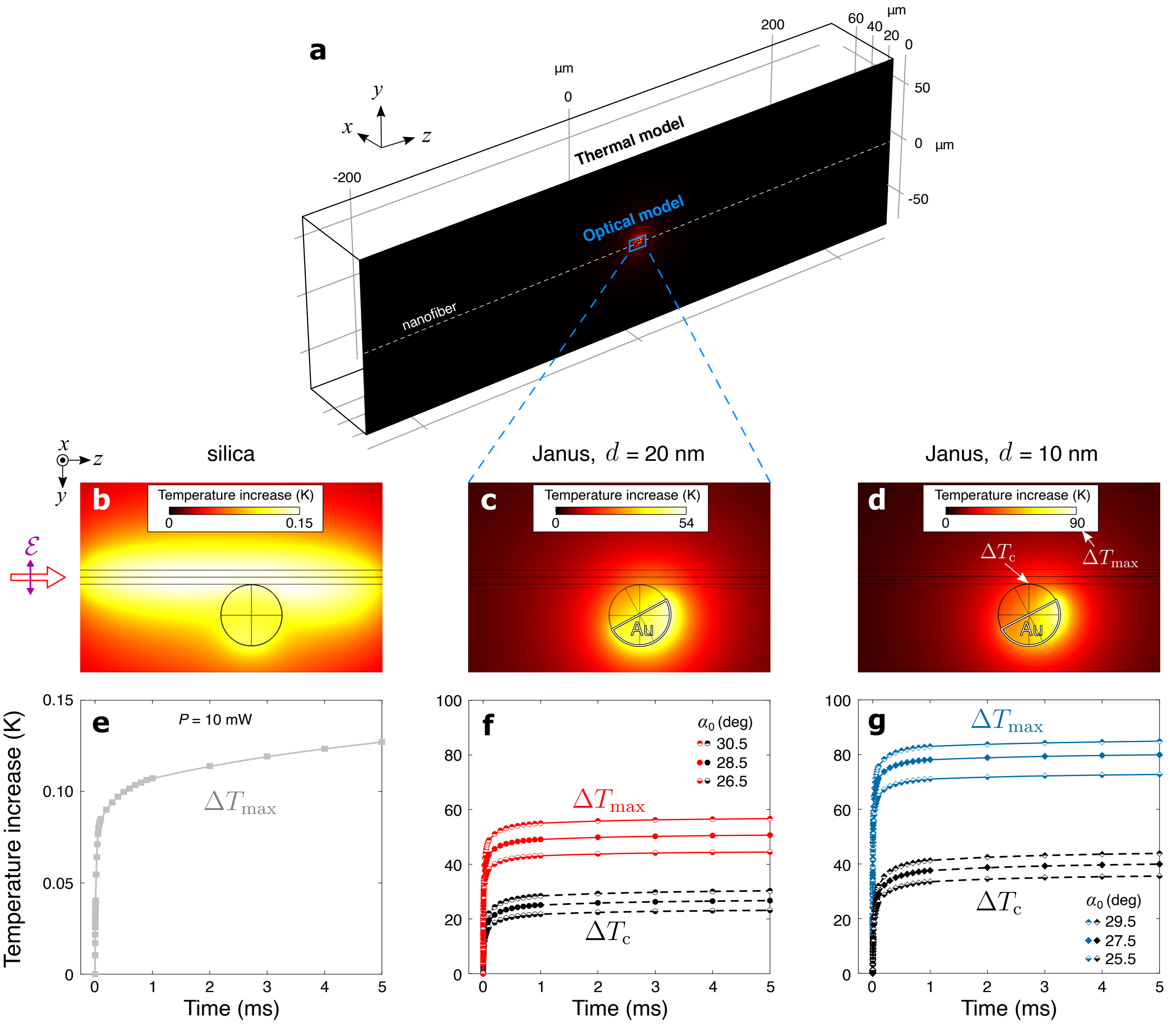

Supplementary Figure 4. **a** Temperature increase, ΔT, in the JP-ONF system simulated for $R_{\mathrm{f}} = 0.35\,\mu\mathrm{m}$, $R_{\mathrm{p}} = 1.5\,\mu\mathrm{m}$, $d = 20$ nm, $\alpha_0 = 28.5°$, $\delta = 10$ nm, $P = 10$ mW, temperature propagation time 10 ms. The blue frame shows the yz-section of the optical simulation domain for comparison. **b, c, d** Spatial distributions of ΔT simulated for 3-µm silica (**b**) and Janus (**c, d**) particles with $d = 20$ nm ($\alpha_0 = 28.5°$) and $d = 10$ nm ($\alpha_0 = 27.5°$). **e, f, g** Temporal evolution of the maximum temperature increase, ΔT_{max}, corresponding to the distributions in **b, c, d**, respectively. Here ΔT_{c} refers to the temperature increase at the particle-nanofiber contact point. Near the experimentally relevant values of α_0, the increase in the gold cap tilt by 1° corresponds to the increase in ΔT by approximately $5 - 7\%$.

Supplementary Note 2. Thermal simulations

Once the optical field distribution in the particle-nanofiber system is found, we can simulate the temperature increase resulting from the conversion of electromagnetic energy into heat and its evolution in time. These simulations are much faster compared to those of light scattering, thus we extended the simulation domain to $(x \times y \times z)_{\mathrm{thermal}} = (200\,\mu\mathrm{m} \times 200\,\mu\mathrm{m} \times 500\,\mu\mathrm{m})$, see Supplementary Figure 4a depicting the distribution of temperature increase, $\Delta T = T - T_0$ (with respect to the room temperature of $T_0 = 20°\mathrm{C} = 273.15$ K), evaluated for a propagation time of 10 ms. Such distributions for 3-µm silica and Janus particles (Supplementary Figure 4b-d) indicate that light-induced heating around an uncoated particle is negligibly small, whereas ΔT reaches 90 K at an input power of 10 mW (notably, the maximum of ΔT is higher for the thinner coating) and exceeds 500 K at 100 mW (Supplementary Figure 5). Assuming the pressure to remain constant, we should expect a cavitation bubble to be created near the gold cap once $\Delta T > 80$ K. However, no cavitation has been seen in our experiments with JPs near an ONF. Such a strong discrepancy between theoretical and experimental results is quite surprising given that the heat transfer model in COMSOL Multiphysics is

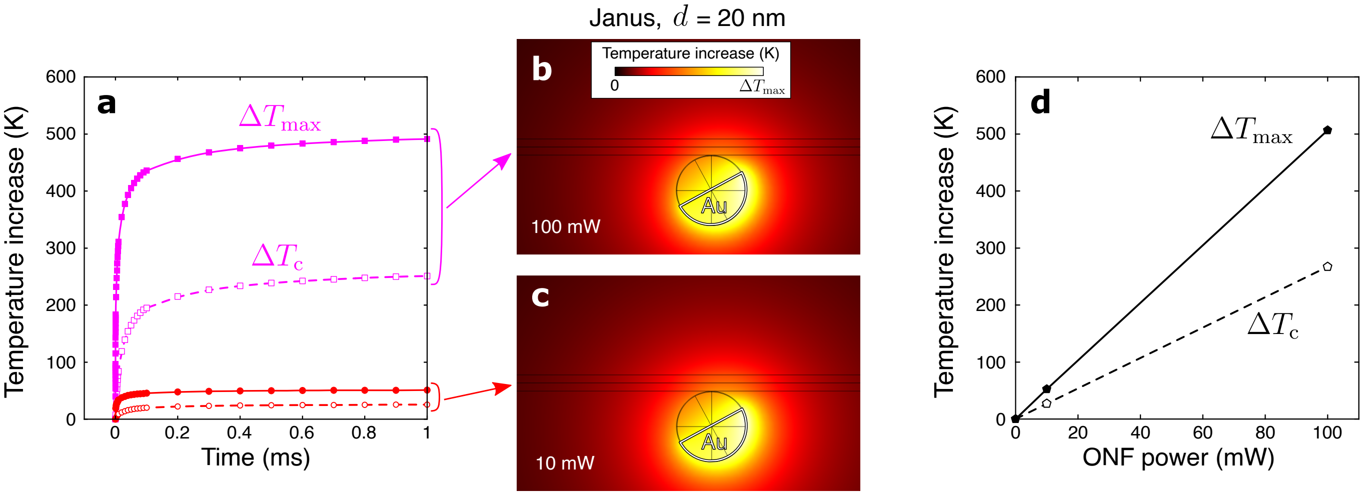

Supplementary Figure 5. **a** Temporal evolution of the temperature increase at the hottest point (filled markers and solid lines) and the particle-nanofiber contact point (empty markers and dashed lines) for a JP with 20-nm-thick gold coating and the optical power values of 100 mW and 10 mW. **b, c** The corresponding spatial distributions of ΔT after 5 ms. **d** Temperature increase as a function of the input optical power. Markers indicate the simulated points, lines in **d** refer to the best linear fit.

the common method applied for analysis of light-induced thermal effects, see for instance [7–9] where good agreement was found between simulations and measurements with laser-illuminated gold films and nanostructures in water.

One possible reason for the overestimation of ΔT in our case is the fact that the geometry in the thermal model is static, whereas in reality the gold cap on a JP only assumes its stable orientation when the particle is being propelled along the nanofiber. In principle, propulsion can be incorporated into the model by iterative displacement of the thermal distribution. However, we argue that such a complication would not be necessary because the particle's motion is much slower compared to the stabilization of the heat distribution. Indeed, as one can see in Supplementary Figure 4f, g, ΔT saturates in less than 0.5 ms, during which time the particle would move by 6.2 nm (according to the linear fit of the experimental points in Fig. 4d of the main text). This displacement corresponds to only 0.2% of the particle's radius, therefore we can safely neglect the JP's propulsion in the thermal model. Notably, the saturation time is virtually independent of the optical power, whereas ΔT at saturation is proportional to the power, as shown in Supplementary Figure 5d.

In order to find a more plausible explanation for the unrealistically high values of ΔT in the simulations, we recall that in our optical model the metallic coating is treated as a perfectly smooth layer of crystalline gold, while in practice the conditions for vapor deposition may vary with the angle between the surface normal and the velocity of the gold atoms. Vapor deposition of gold on silica substrates without any special treatment (as in our experiments) is known to produce films with nanoscale imperfections [10], which can only get more prominent as the surface deviates from a plane. Moreover, particles that happened to be tightly packed during the coating have noticeable defects at the edges of the gold cap (see Fig. 1a, b of the main text). Therefore, the actual properties of the material near the edges of the gold cap on a JP may significantly differ from those assumed in the numerical simulations. In particular, obstructions to the propagation of surface plasmons in these areas would lead to an increase of scattering and the reduction of absorption of light, hence the actual light-induced heating might be much smaller than that calculated for a perfect golden film. Due to the size limitations of our 3D model, we cannot include nanoscale imperfections of the coating. Instead, we tried to mimic the lack of absorption by simply removing gold from the areas located further than $R_{\rm cap} \leq R_{\rm p}$ from the symmetry axis of the particle. As shown in Fig.7 of the main text, a reduction of the effective radius of the gold cap, $\tilde{R} = R_{\rm cap}/R_{\rm p}$, by a few percent would result in a dramatic decrease of ΔT (as well as the optical forces, F_z and F_y, and the propulsion enhancement, ξ_z) to values below the boiling temperature of water. Importantly, the reduction of $\tilde{R}$ would also affect the spatial distribution of ΔT (compare Fig. 7a and Fig. 7b in the main text) and thus the temperature gradient, ∇T. Without the knowledge of these distributions we cannot accurately calculate thermal forces and torques on a JP near an ONF. One should also note that such calculations rely on surface integration of the fluid slip velocity, $\mathbf{v} = D_T \nabla_{\parallel} T$, where $\nabla_{\parallel} T$ is the temperature gradient tangential to the surface, and D_T is the thermodiffusion coefficient which is a function of multiple parameters of the particle-fluid system [11]. Evaluation of D_T for our experimental conditions is a very challenging task that is beyond the scope of the present study.

Supplementary Note 3. Particle tracking

Each video was processed by custom MATLAB codes (see the Code Availability statement in the main text) based on the function below

```
function [t,posX,posY] = particle_track(fname,steps,R)
% INPUT:
%   fname - name of the video file to read
%   steps - number of frames at which the particle must be found
%   R - approximate radius of the particle, in pixels
% OUTPUT:
%   t - time vector for the analyzed frames
%   posX, posY - vectors of the particle’s X and Y coordinates
readerObj = VideoReader(fname);
NumOfFrames = floor(readerObj.Duration*readerObj.FrameRate);
t = readerObj.Duration*[0:1/steps:(1-1/steps) 1-1/NumOfFrames];
for i=1:length(t)
     readerObj.CurrentTime = t(i);
     frame = rgb2gray(readFrame(readerObj));
     frameInv = max(frame(:))-frame;
     center = imfindcircles(frameInv,R,’Sensitivity’,0.995);
     posX(i) = center(1,1);
     posY(i) = center(1,2);
end
```

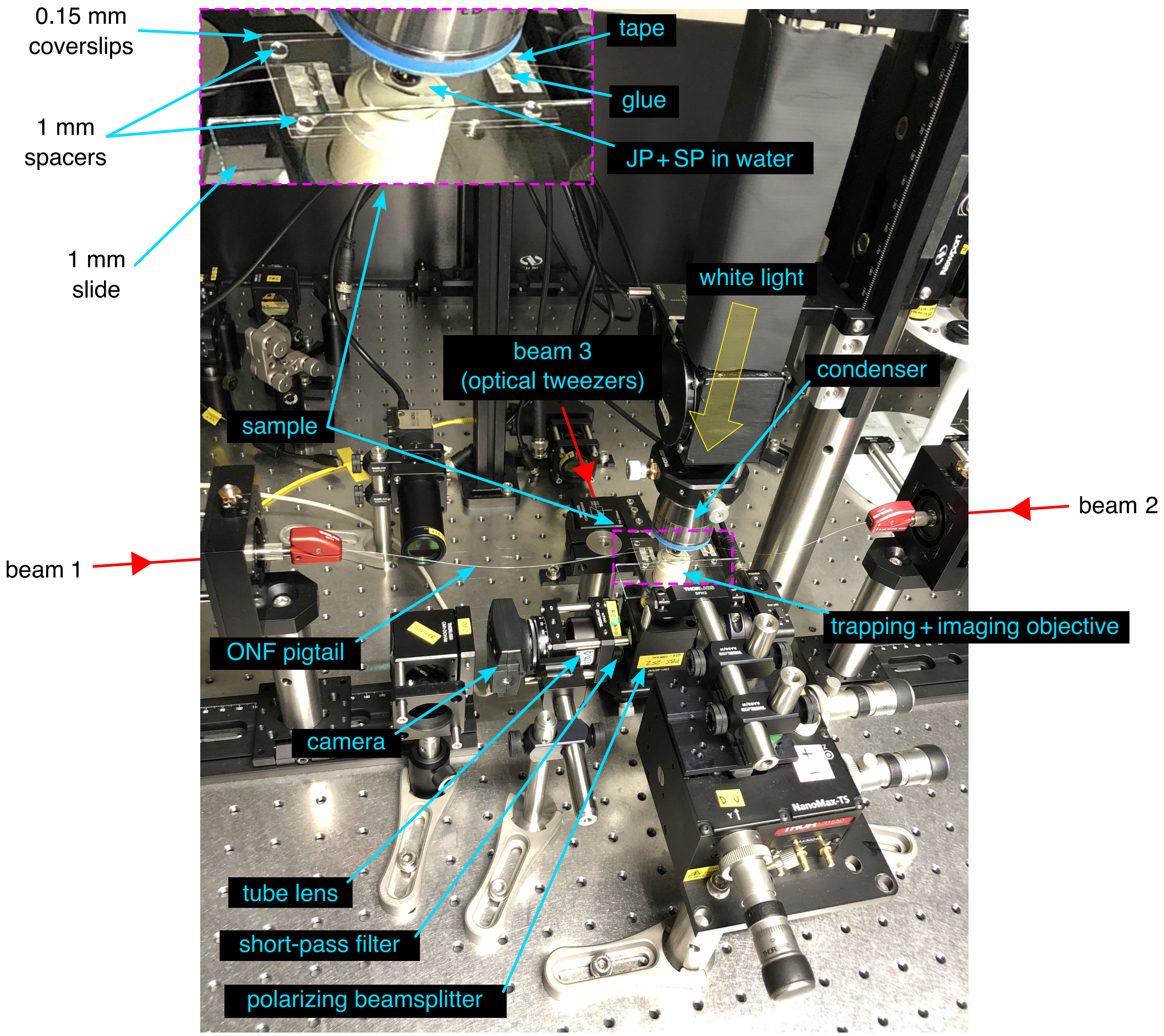

Supplementary Figure 6. **Experimental setup in detail.** Note: this is an older version of the imaging arm, in the final version a higher-quality CMOS camera (DCC3240C instead of DCC1545M, both by Thorlabs, Inc.) and a longer-focus tube lens (achromatic doublet AC254-075-A-ML instead of AC254-050-A-ML, Thorlabs) were used.

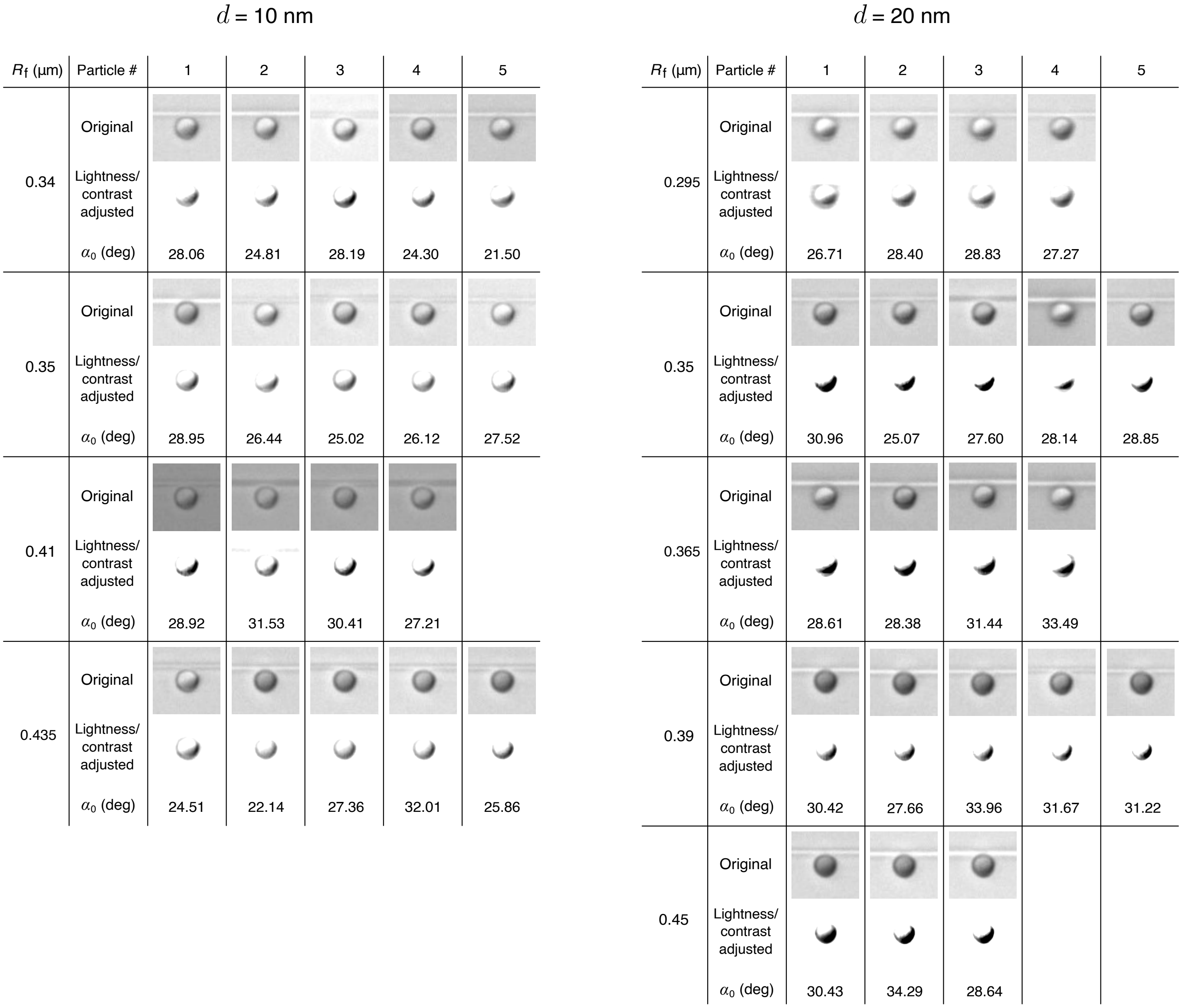

Supplementary Figure 7. **Measurements of the gold cap orientation angle.** The gold cap orientation can be directly seen when the JP is propelled under horizontal ($\mathcal{E} \parallel \mathbf{y}$) polarization of the beam coupled to the ONF. To measure the orientation angle, α_0, we extract a random video frame from the middle of the recorded track (in order to have the particle illuminated symmetrically). The frame is imported into Inkscape (an open-source graphics software) where we increase the contrast (by 20% for $d = 10$ nm and 40% for $d = 20$ nm) and lightness (0-10% and 10-20%, respectively) of the image, and then determine the angle using the Measurement Tool. In order to improve the accuracy of these measurements, we repeated them for 3 to 5 different JPs per experimental sample. We also tried propelling some particles by beam 2 (towards $z < 0$) and found that the gold cap orientation with respect to the propagation direction was preserved, thereby confirming that the illumination was indeed uniform. The above images correspond to the experimental data in Fig. 5c of the main text.

---

[1] J. P. Barton, D. R. Alexander, and S. A. Schaub, "Theoretical determination of net radiation force and torque for a spherical particle illuminated by a focused laser beam," J. Appl. Phys. **66**, 4594–4602 (1989).

[2] K. Y. Bliokh, A. Y. Bekshaev, and F. Nori, "Extraordinary momentum and spin in evanescent waves," Nat. Commun. **5**, 1–8 (2014).

[3] G. Tkachenko, I. Toftul, A. Vylegzhanin, V. G. Truong, M. Petrov, and S. Nic Chormaic, "Detection of the transverse spin of light by twisting anisotropic particles near an optical nanofiber waveguide," Proc. SPIE, Optical Manipulation and Structured Materials Conference **11926**, 69–71 (2021).

[4] K. Y. Bliokh and F. Nori, "Transverse and longitudinal angular momenta of light," Phys. Rep. **592**, 1–38 (2015).

[5] K. H. Chowdhury, N. Soyaib, and S. Mia, "Comparison of sliding frictions of different materials using a digital sliding friction tester," Proc. International Conference on Mechanical, Industrial and Energy Engineering **ICMIEE-PI-140425** (2014).
[6] F. Charru, E. Larrieu, J.-B. Dupont, and R. Zenit, "Motion of a particle near a rough wall in a viscous shear flow," J. Fluid Mech. **570**, 431–453 (2007).
[7] B. J. Roxworthy, A. M. Bhuiya, S. P. Vanka, and K. C. Toussaint, "Understanding and controlling plasmon-induced convection," Nat. Commun. **5**, 3173 (2014).
[8] Q. Jiang, B. Rogez, J.-B. Claude, G. Baffou, and J. Wenger, "Temperature measurement in plasmonic nanoapertures used for optical trapping," ACS Photonics **6**, 1763–1773 (2019).
[9] Y. Kim, H. Ding, and Y. Zheng, "Investigating water/oil interfaces with opto-thermophoresis," Nat. Commun. **13**, 3742 (2022).
[10] R. Malureanu and A. Lavrinenko, "Ultra-thin films for plasmonics: A technology overview," Nanotechnol. Rev. **4**, 259–275 (2015).
[11] A. Würger, "Thermophoresis in colloidal suspensions driven by Marangoni forces," Phys. Rev. Lett. **98**, 138301 (2007).
