# Supplementary material for: Evanescent field trapping and propulsion of Janus particles along optical nanofibers
Source: Nat Commun. 2023 Mar 27;14:1691. doi: 10.1038/s41467-023-37448-2 (PMC10043011; doi:10.1038/s41467-023-37448-2)
Supplement: Supplementary file 3 — Description of Additional Supplementary Files [file 41467_2023_37448_MOESM3_ESM.pdf]

Description of Additional Supplementary Files

File Name: Supplementary Movie 1
Description: Demonstration of light-induced trapping and propulsion of a silica-gold Janus particle (diameter 3 um, coating thickness 20 nm) in the evanescent field of a single-mode optical nanofiber (ONF1, diameter 700 nm) coupled to counterpropagating laser beams 1 and 2. The particle is brought in contact with the waist region of the nanofiber by means of low-power optical tweezers (beam 3).

File Name: Supplementary Movie 2
Description: Typical light-induced propulsion of silica and Janus particles near an optical nanofiber. Beam 1 is coupled to the vertically (perpendicularly to the image plane) polarized fundamental mode propagating from left to right. The transmitted power is 80 mW for all 4 panels. Janus particles with both 10- and 20-nm thick coating always move faster than uncoated particles of the same size.

File Name: Supplementary Movie 3
Description: Comparison between light-induced propulsion of Janus particles and silica particles under the action of horizontally (parallel to the image plane) polarized mode propagating from left to right. At this polarization, the particles are trapped in the image plane, and the orientation of the gold cap on Janus particles can be measured. Such videos were also used for evaluation of the optical trapping stiffness in the radial direction.
